# Clinical trials of immunotherapy in triple-negative breast cancer

**DOI:** 10.1007/s10549-022-06665-6

**Published:** 2022-07-14

**Authors:** Frederick M. Howard, Alexander T. Pearson, Rita Nanda

**Affiliations:** Section of Hematology/Oncology, Department of Medicine, University of Chicago Medicine & Biological Sciences, 5841 S. Maryland Ave MC 2115, Chicago, IL 60637 USA

**Keywords:** Triple-negative breast cancer, Immunotherapy, Checkpoint blockade, Clinical Trials, Atezolizumab, Pembrolizumab

## Abstract

**Purpose:**

Immunotherapy has started to transform the treatment of triple-negative breast cancer (TNBC), in part due to the unique immunogenicity of this breast cancer subtype. This review summarizes clinical studies of immunotherapy in advanced and early-stage TNBC.

**Findings:**

Initial studies of checkpoint blockade monotherapy demonstrated occasional responses, especially in patients with untreated programmed death-ligand 1 (PD-L1) positive advanced TNBC, but failed to confirm a survival advantage over chemotherapy. Nonetheless, pembrolizumab monotherapy has tumor agnostic approval for microsatellite instability-high or high tumor mutational burden cancers, and thus can be considered for select patients with advanced TNBC. Combination chemoimmunotherapy approaches have been more successful, and pembrolizumab is approved for PD-L1 positive advanced TNBC in combination with chemotherapy. This success has been translated to the curative setting, where pembrolizumab is now approved in combination with neoadjuvant chemotherapy for high-risk early-stage TNBC.

**Conclusion:**

Immunotherapy has been a welcome addition to the growing armamentarium for TNBC, but responses remain limited to a subset of patients. Innovative strategies are under investigation in an attempt to induce immune responses in resistant tumors—with regimens incorporating small-molecule inhibitors, novel immune checkpoint targets, and intratumoral injections that directly alter the tumor microenvironment. As the focus shifts toward the use of immunotherapy for early-stage TNBC, it will be critical to identify those who derive the most benefit from treatment, given the potential for irreversible autoimmune toxicity and the lack of predictive accuracy of PD-L1 expression in the early-stage setting.

## Introduction

Triple-negative breast cancer (TNBC) is defined by the absence of estrogen and progesterone receptor expression and lack of human epidermal growth factor receptor 2 (HER2) amplification. This receptor profile accounts for approximately 15% of breast cancer cases diagnosed in the USA, and more frequently afflicts young women, those of African and Hispanic ancestry, and those who harbor a deleterious mutation in *BRCA1* [1]. Early-stage TNBC is associated with a higher risk of recurrence compared with other subtypes, and advanced stage disease has a poor prognosis with a median survival of approximately 18 months (mos) [2]. While endocrine and HER2-directed therapies have improved prognosis of other breast cancer subtypes, until recently, targeted agents remained elusive for the treatment of TNBC. By developing trials specifically for advanced TNBC, we have finally begun to see improvements in outcomes. The PARP inhibitors olaparib and talazoparib are FDA approved for BRCA-associated advanced TNBC based on the findings of the OlympiAD and EMBRACA randomized phase III trials [3, 4]. In both trials, treatment with a PARP inhibitor resulted in meaningful improvements in progression-free survival (PFS) and patient-reported quality of life outcomes over physician’s choice of chemotherapy. And, the Trop-2 targeting antibody–drug conjugate sacituzumab govitecan gained approval for advanced TNBC based on the randomized phase 3 ASCENT trial [5]. In ASCENT, treatment with sacituzumab govitecan resulted in an impressive 3.7 mo improvement in median PFS and 5.4 mo improvement in median overall survival (OS) compared to physician’s choice chemotherapy. Additionally, pembrolizumab has gained approval for programmed death-ligand 1 (PD-L1) positive (PD-L1 +) advanced TNBC, and for early-stage high-risk TNBC in combination with standard neoadjuvant chemotherapy. However, as immunotherapy moves into the early-stage curative setting, given the potential for irreversible immune-related toxicities there is an increasing need to identify predictive biomarkers of response and/or toxicity, so we can fully realize the potential of precision immunotherapy.

### Advanced triple-negative breast cancer

Early immunotherapy approaches in breast cancer included the use of interferon and interleukins, with disappointing efficacy and an unacceptable toxicity profile [6, 7]. The success of checkpoint blockade in melanoma prompted the study of these agents in a variety of advanced solid malignancies, including breast cancer. Early clinical trials of PD-1/PD-L1 inhibitors in breast cancer focused on TNBC, as TNBC has a higher level of tumor infiltrating lymphocytes (TILs) [8–10] and PD-L1 expression as compared to other breast cancer subtypes, suggesting that a subset of TNBCs are immunogenically active [11].

#### Checkpoint inhibitor monotherapy

Initial nonrandomized trials of checkpoint inhibitor monotherapy provided evidence of single agent activity in TNBC, particularly in tumors which have PD-L1 expression and a high level of TILs (Table 1) [12–16]. KEYNOTE-012 was the first study to demonstrate proof of concept that checkpoint blockade was an effective strategy for the treatment of advanced TNBC [13]. In the study, 32 women with PD-L1 positive disease (defined as stromal expression ≥ 1%) received pembrolizumab monotherapy. Among 27 evaluable patients, an overall response rate (ORR) of 18.5% was documented. A multicohort phase I trial evaluated the safety and efficacy of atezolizumab monotherapy in metastatic TNBC with at least 5% PD-L1 expression in tumor infiltrating immune cells (IC), later expanding to include patients with PD-L1- disease [12]. The ORR was 10% in 115 evaluable patients; 24% in the 21 patients treated in the first line metastatic setting, and 6% in the 94 patients treated in the second line and beyond. No patients with PD-L1- disease responded, whereas the ORR was 12% in the PD-L1 + group. Tumor cell (TC) PD-L1 positivity did not discriminate between responders and non-responders. The multicohort phase 1b JAVELIN solid tumor study evaluated avelumab monotherapy in patients with metastatic breast cancer of any subtype. Responses were enriched in patients with TNBC (ORR 5.5%) compared to other subtypes, especially among those with PD-L1 positive TNBC (ORR 22.2%).

**Table 1:** Non-randomized clinical trials of immune checkpoint blockade with or without chemotherapy in metastatic TNBC

Trial	Key inclusion	Treatment	Subgroups	Sample size	ORR (%)	DCR (%)	Median DOR (months)	Median PFS (months)	Median OS (months)
NCT01772004^a^	Any line	Avelumab		58	5.2	31	NR	5.9	9.2
JAVELIN Solid Tumor									
NCT01375842	Any line	Atezolizumab		115	10	13	21.0	1.4	8.9
			PD-L1 IC ≥ 1%	91	12	15	21.0	1.4	10.1
			PD-L1 IC < 1 %	21	0	5	N/A	1.4	6.0
NCT01848834	Any line	Pembrolizumab		32	18.5	25.9	NR	1.9	11.2
	PD-L1 IC/TC								
	≥ 1%								
KEYNOTE-012									
NCT02447003	≥ 2nd line	Pembrolizumab		170	5.3	7.6	NR	2.0	9.0
KEYNOTE-086									
Cohort A									
			PD-L1 CPS ≥ 1	105	5.7	9.5	NR	2.0	8.8
			PD-L1 CPS < 1	64	4.7	4.7	4.4	1.9	9.7
NCT02447003	1st line	Pembrolizumab		84	21.4	23.8	10.4	2.1	18.0
	PD-L1 CPS ≥ 1								
KEYNOTE-086									
Cohort B									
NCT01633970	Any line	Atezolizumab +		33	39.4	51.5	9.1	5.5	14.7
		*Nab*-paclitaxel							
			PD-L1 IC ≥ 1%	12	41.7	91.7	9.1	6.9	21.9
			PD-L1 IC < 1%	12	33.3	75.0	10.2	5.1	11.4
NCT02513472	1st line	Pembrolizumab + eribulin	PD-L1 CPS ≥ 1	29	34.5	NA	8.3	6.1	21.0
KEYNOTE-150									
			PD-L1 CPS < 1	31	16.1		15.2	3.5	15.2
	2nd or 3rd line		PD-L1 CPS ≥ 1	45	24.4	NA	8.2	4.1	14.0
			PD-L1 CPS < 1	44	18.2		8.6	3.9	15.5
NCT03044730^a^	Any line	Pembrolizumab + capecitabine		15	13	60	NA	4.0	15.3
NCT03121352	1st or 2nd line	Pembrolizumab + carboplatin +		30	52	77.8	NA	6.1	11.5
		*Nab*-paclitaxel							

*ORR* overall response rate, *DCR* disease control rate, *DOR* duration of response, *PFS* progression-free survival, *OS* overall survival, *PD-L1* programmed death-ligand 1, *IC* immune cell, *TC* tumor cell, *CPS* combined positive score, *NR* not reached, *NA* not available

^a^Data from the TNBC subgroup of included patients is listed

KEYNOTE-086 further highlighted the impact of prior lines of therapy on response to checkpoint blockade [14, 15]. Cohort A of the trial enrolled 170 patients with previously treated metastatic TNBC and demonstrated an ORR of 5.3% in the overall population; the ORR was 5.7% in patients with PD-L1 + tumors, and 4.7% in those with PD-L1- disease. PD-L1 positivity was defined as CPS (combined positive score) ≥ 1 using the Dako 22C3 assay—with CPS calculated as the total number of PD-L1 staining cells divided by viable tumor cells, multiplied by 100. Cohort B enrolled 84 patients with PD-L1 + untreated advanced disease, and demonstrated an ORR of 21.4%; the response rate approached 40% in those who had tumors characterized by a high level of stromal TILs. The greater response in previously untreated patients may be due immune exhaustion or changes in the tumor immune microenvironment with subsequent lines of chemotherapy [17].

While these initial studies of monotherapy reported modest response rates, the durability of response was quite remarkable, and longer than what is typically seen with chemotherapy. Thus, the KEYNOTE-119 trial formally compared checkpoint inhibitor monotherapy to chemotherapy, randomizing 622 patients with advanced TNBC to either pembrolizumab or treatment of physician’s choice (capecitabine, eribulin, gemcitabine, or vinorelbine, Table 2) [18]. Included patients were allowed 1–2 prior lines for therapy in the advanced stage setting. The trial failed to meet its primary and secondary endpoints of OS and PFS in three pre-specified categories (CPS ≥ 10, CPS ≥ 1, and the ITT population). Increasing response rates were observed with increasing levels of PD-L1 positivity; ORR with pembrolizumab was 9.6% in the ITT population, 12.3% in the CPS ≥ 1 group, and 17.7% in the CPS ≥ 10 group. Median duration of response was numerically longer with pembrolizumab as compared to chemotherapy (12.2 mos vs 8.3 mos, not statistically tested) in the ITT population. An exploratory analysis found improved OS in the subgroup of approximately 17% of patients with CPS ≥ 20 (median 14.9 mos vs 12.5 mos, not statistically tested), although the significance of this finding is limited by the post hoc nature of the analysis. Liver metastases frequently have lower infiltration with inflammatory cells, but exploratory analyses by site of metastases demonstrated similar magnitudes of benefit with pembrolizumab versus chemotherapy at each CPS cutoff, regardless of liver or lung involvement [19]. Rates of grade 3–5 adverse events were lower with pembrolizumab than with chemotherapy (14% vs 36%, not statistically tested); health related quality of life (HRQoL) outcomes also favored pembrolizumab, most notably in the CPS ≥ 10 cohort, where patients reported lower scores on systemic therapy side effects and nausea/vomiting scales [20].

**Table 2:** Randomized clinical trials of immunotherapy in advanced triple-negative breast cancer

Trial	Key inclusion	Treatment arms	Subgroups	Sample size		ORR (%)		Median PFS (months)		Median OS (months)	
				IN	CT	IN	CT	IN	CT	IN	CT
NCT02425891	1st line	Atezolizumab + *nab-*paclitaxel	ITT	451	451	56	46	7.2	5.5	21.0	18.7
IMpassion130	DFI ≥ 12 months	Placebo + *nab*-paclitaxel	PD-L1 IC ≥ 1%	185	184	59	43	7.5	5.0	25.0	18.0
NCT03125902	1st line	Atezolizumab + paclitaxel	ITT	431	220	54	47	5.7	5.6	19.2	22.8
IMpassion131	DFI ≥ 12 mont hs	Placebo + paclitaxel	PD-L1 IC ≥ 1%	191	101	63	55	6.0	5.7	22.1	28.3
NCT02555657	2nd or 3rd line	Pembrolizumab	ITT	312	310	9.6	10.6	2.1	3.3	9.9	10.8
		Capecitabine, eribulin,	PD-L1 CPS ≥ 1	203	202	12.3	9.4	2.1	3.1	10.7	10.2
		Gemcitabine, or vinorelbine	PD-L1 CPS ≥ 10	96	98	17.7	9.2	2.1	3.4	12.7	11.6
KEYNOTE-119	Prior anthracycline and taxane										
			PD-L1 CPS ≥ 20	57	52	26.3	11.5	3.4	2.4	14.9	12.5
KEYNOTE-355	1st line	Pembrolizumab +	ITT	566	281	40.8	37.0	7.5	5.6	17.2	15.5
		*Nab*-paclitaxel, paclitaxel, or gemcitabine/carboplatin	PD-L1 CPS ≥ 1	425	211	44.9	38.9	7.6	5.6	17.6	16.0
		Placebo +	PD-L1 CPS ≥ 10	220	103	52.7	40.8	9.7	5.6	23.0	16.1
NCT02819518	DFI ≥ 6 months	*Nab*-paclitaxel, paclitaxel, or gemcitabine/carboplatin									
NCT02299999^a^	1st or 2nd line	Maintenance durvalumab	ITT	47	35	NA		HR	HR	21	14
SAFIR02-BREAST IMMUNO	CR/PR/SD after 6–8 cycles chemo	Maintenance chemotherapy						0.87	1.00		
	No actionable mutation										
NCT02708680	> 2nd line	Atezolizumab + entinostat	ITT	40	41	10.0	2.4	1.68	1.51	9.8	12.4
ENCORE-602											
		Atezolizumab + placebo									
NCT02322814	1st line	Paclitaxel + cobimetinib	ITT	47	43	38.3	20.9	5.5	3.8	16.0	19.6
		Paclitaxel + placebo									
COLET											
Cohort I											
NCT02322814^b^	1st line	Atezolizumab + cobimetinib + *nab-*paclitaxel	ITT	31	32	29.0	34.4	7.0	3.8	NR	11.0
COLET		Atezolizumab + cobimetinib + paclitaxel									
Cohorts II/III											

Intervention arm regimen listed followed by control arm for each trial

*ORR* overall response rate, *PFS* progression-free survival, *OS* overall survival, *IN* intervention, *CT* Control, *DFI* disease-free interval, *ITT* intention to treat, *PD-L1* programmed death-ligand 1, *IC* immune cell, *CPS* combined positive score, *HR* hazard ratio, *NA* not available, *NR* not reached

^a^Data from the triple-negative subgroup of included patients is listed

^b^Since there was no placebo arm in the second randomization of COLET, data for the *nab*-paclitaxel arm is listed under the intervention columns, and data for paclitaxel arm is listed under the control columns

The phase II SAFIR02-BREAST IMMUNO trial evaluated the use of checkpoint inhibitor monotherapy as a maintenance treatment, comparing the anti-PD-1 antibody durvalumab to continued chemotherapy in metastatic HER2-negative breast cancer patients who did not progress after 6–8 cycles of chemotherapy, and did not have actionable genetic mutations. The primary endpoint of PFS favored chemotherapy in the overall study population, but in an exploratory analysis of the 82 patients with TNBC, durvalumab maintenance was associated with a trend toward improved PFS (HR 0.87; 95% CI 0.54–1.42) and significantly improved OS (median 21.2 mos vs 14.0 mos; HR 0.54, *p* = 0.0377) [21]. This benefit was more pronounced in TNBC patients with PD-L1 IC ≥ 1%, or CD274 (encoding PD-L1) gene gain/amplification. Given the approval of pembrolizumab with chemotherapy in PD-L1 positive TNBC, it is unlikely that maintenance with checkpoint inhibitors will have a role outside of continuation of treatment in patients eligible for frontline immunotherapy.

Although there is currently no breast cancer specific indication for pembrolizumab monotherapy, there are tumor agnostic indications for patients with high TMB (≥ 10 mut/Mb) and microsatellite instability-high (MSI-H) status. These indications were granted due to the results of the multicohort phase II KEYNOTE-158 study, evaluating biomarkers predictive of benefit of pembrolizumab monotherapy in advanced solid tumors [22, 23]. Breast cancers were underrepresented in KEYNOTE-158, with only five patients included in the MSI-H analysis, and none included in the TMB analysis. However, among the 253 patients in KEYNOTE-119 where TMB status was available and TMB was ≥ 10 mut/Mb (*n* = 14), there was a trend toward improved response with pembrolizumab versus chemotherapy (ORR 14% vs 8%, not statistically tested) [24]. Only a small fraction of TNBC patients will meet these indications, but monotherapy remains a reasonable consideration in eligible patients, recognizing response rates may be lower than combination with chemotherapy.

#### Combination of checkpoint inhibitors with chemotherapy

Although checkpoint inhibitor monotherapy led to durable responses in a subset of patients, the low rates of response prompted consideration of combination regimens. Additionally, chemotherapy may have immunomodulatory benefits—including a reduction of regulatory T cells [25], induction of a type I interferon response due to tumor antigen release [26], and increased expression of PD-L1 [27].

A phase 1b trial of atezolizumab and *nab*-paclitaxel demonstrated responses in over one third TNBC patients with an acceptable safety profile [28], and paved the way for this approach to be explored in the phase III IMpassion130 trial. IMpassion130 randomized 902 patients with untreated advanced TNBC to receive *nab*-paclitaxel with either atezolizumab or placebo in a 1:1 ratio (Table 2) [29]. Patients with prior therapy for early breast cancer were eligible, but must have had at least a 12-month disease-free interval (DFI). PD-L1 IC status was assessed with the Ventana SP142 assay, but positivity was not required for enrollment; PD-L1 + disease was defined as at least 1% IC positive for PD-L1 expression. Primary outcome measures included OS and PFS, assessed in all randomized patients as well as the PD-L1 + subgroup. PFS was significantly longer with atezolizumab in both the intention to treat (ITT) population [median 7.2 mos vs 5.5 mos; hazard ratio (HR) 0.80, *p* = 0.002] and the PD-L1 + subgroup (median 7.5 mos vs 5.0 mos; HR 0.62, *p* < 0.001). The OS was not statistically better with the addition of atezolizumab in the ITT population. A clinically meaningful 7 mo improvement in median OS was seen with atezolizumab in the PD-L1 + subgroup, but formal significance testing was not performed due to the pre-specified hierarchical statistical analysis plan [30]. Immune related thyroid dysfunction and pneumonitis occurred in 17.3% and 3.1% of patients in the atezolizumab arm. No clinical meaningful differences in HRQoL or patient-reported treatment symptoms (fatigue, diarrhea, nausea/vomiting) were seen between treatment arms [31]. Further biomarker analysis demonstrated prolonged PFS with PD-L1 TC positivity, intratumoral CD8 positivity, and with ≥ 10% stromal TILs—although the former two factors correlated with PD-L1 IC positivity. Post hoc analysis also demonstrated that the preponderance of patients who are PD-L1 IC positive by the Ventana SP142 assay are also positive by the Dako 22C3 (CPS ≥ 1) and Ventana SP263 (IC ≥ 1%) assays, but treatment benefit was most pronounced in those positive by the Ventana SP142 assay [32].

Although atezolizumab received accelerated FDA approval for advanced PD-L1 positive TNBC based on IMpassion130, further data have led to the withdrawal of this approval. The similarly designed phase III IMpassion131 trial randomized 651 patients with untreated advanced TNBC to paclitaxel with or without atezolizumab in a 2:1 ratio [33]. The primary endpoint of PFS was not met in the PD-L1 + subgroup (median 6.0 mos vs 5.7 mos; HR 0.82, *p* = 0.20) and or the ITT population (median 5.7 mos for both groups; HR 0.86, p not formally tested). Median OS was also numerically longer in the placebo arms in both the PD-L1 + and ITT populations (22.1 mos vs 28.3 mos, and 19.2 mos versus 22.8 mos, respectively), however, the study was not powered for this endpoint and survival data are immature. Exposure to paclitaxel was similar in both treatment arms, and the toxicity profile was similar to IMpassion130; the reason for the lack of benefit from atezolizumab is unclear. While it is possible that the steroid premedication with paclitaxel could dampen immune responses or the albumin bound formulation of paclitaxel may have more potent immunomodulatory effects [34], this seems unlikely as the selection of paclitaxel versus *nab*-paclitaxel has and not influenced results in other trials. Unmeasured confounders may have led to an imbalance between the two treatment arms, masking any benefit in the atezolizumab arm. Further study is needed to clarify the discrepancies in these two trials, and the role (if any) of atezolizumab in advanced TNBC.

More definitive success of immunotherapy for advanced TNBC was seen in the phase III KEYNOTE-355 trial, which randomized 847 patients with untreated metastatic or inoperable locally recurrent TNBC in a 2:1 ratio to chemotherapy (investigator’s choice of *nab*-paclitaxel, paclitaxel, or gemcitabine/carboplatin) with pembrolizumab or with placebo [35]. Participants must have had a DFI of at least 6 mos from the completion of curative chemotherapy. Primary endpoints were PFS and OS in the ITT, PD-L1 CPS ≥ 1, and CPS ≥ 10 populations. An increase in PFS was seen with the addition of pembrolizumab in all three groups, but with adjustments for multiple hypothesis testing, significant benefit was only seen in the CPS ≥ 10 subgroup (PFS 9.7 mos vs 5.6 mos; HR 0.65, *p* = 0.0012). Overall response rate favored the addition of pembrolizumab in patients with CPS ≥ 10 (52.7% vs 40.8%, not statistically tested), and median duration of response was an impressive 19.3 mos in this population (vs 7.3 mos with placebo) [36, 37]. OS was also prolonged in the CPS ≥ 10 population (HR 0.73,* p* = 0.0093) [37], and rates of grade 3–5 adverse events were similar in both treatment groups. Immune related adverse events (irAEs) were consistent with pembrolizumab monotherapy, with the most frequent serious (grade 3 +) adverse events being skin reactions (2%) and pneumonitis (1%). Interestingly, as compared to the IMpassion131 trial, subgroup analysis suggested that patients treated with either paclitaxel or *nab*-paclitaxel benefitted from the addition of immunotherapy. Based on these data, pembrolizumab was granted approval by the FDA in combination with chemotherapy for patients with PD-L1 + (CPS ≥ 10) TNBC. Early phase clinical trials have also described the safety and efficacy of pembrolizumab in combination with other chemotherapy regimens in the treatment of metastatic TNBC, including eribulin, capecitabine, and the combination of carboplatin and *nab*-paclitaxel (Table 1) [38–40]. Evaluation of the efficacy of pembrolizumab in combination with the antibody–drug conjugate sacituzumab govitecan is being investigated for PD-L1-TNBC in the randomized phase II Saci-IO TNBC trial (NCT04468061) [41].

#### Early phase trials of novel immunotherapy combinations

The addition of a checkpoint inhibitor to a standard chemotherapeutic regimen for.

PD-L1 + TNBC can clearly improve patient outcomes. However, several small studies have evaluated the use of limited low-dose chemotherapy prior to immunotherapy treatment, given the impact of chemotherapy on the tumor microenvironment [25, 26, 42]. A phase II trial evaluating a single priming dose of cyclophosphamide one day prior to initiation of pembrolizumab in previously treated metastatic TNBC demonstrated an ORR of 21% with a median PFS of 1.8 mos and median OS of 6.3 mos [43]. Although responses rates were higher than the previously treated cohort of the KEYNOTE-086 trial, OS was comparable, and cyclophosphamide failed to reduce Tregs on correlative analysis. The phase II TONIC trial evaluated no induction or a short course of induction therapy with cyclophosphamide, cisplatin, doxorubicin, or 24 Gy radiation prior to treatment with the anti-PD-1 antibody nivolumab in 70 patients with 3 or fewer prior lines of treatment for metastatic TNBC [44]. The ORR was 20%, with the highest response rate seen with doxorubicin induction (ORR 35%)—however, this cohort was also enriched for previously untreated patients. The doxorubicin and cisplatin (ORR 23%) arms both demonstrated trends toward increased T-cell infiltration and upregulation of inflammatory gene signatures after induction treatment, supporting the immunomodulatory role of the chemotherapy partner administered with immunotherapy.

Like chemotherapy, radiation has multiple immunomodulatory effects, including production of neoantigens, presentation of tumor antigens to immune effector cells, upregulation of PD-L1, and stimulation of a type I interferon signal in tumors with an increase in T-cell infiltration [45–47]. This induction of anti-tumor immunity can lead to a response in non-irradiated sites of disease, termed the abscopal effect [45]. The ORR in the radiation induction cohort of the TONIC trial was only 8%, and not suggestive of dramatic synergy between radiation therapy and immunotherapy. In another phase II trial, a total dose of 30 Gy of radiotherapy was delivered in 5 daily fractions in 17 patients with TNBC who had received a median of 3 lines (range of 0–7) of prior therapy [48]. Pembrolizumab was administered within 3 days of the first fraction of radiation. A response was documented in 3 patients (17.6%), all of whom experienced a complete response with durations ranging from 20 to 108 weeks, and no patients discontinued treatment due to toxicity. Further study is needed to identify the optimal dose and timing of radiation in concert with immunotherapy for optimal synergy.

Other immune checkpoint inhibitors aside from those targeting PD-1 or PD-L1 have been studied in TNBC. Cytotoxic T lymphocytic antigen 4 (CTLA-4) was the first clinically targeted immune checkpoint receptor, but studies in TNBC are limited. A pilot study of durvalumab with the CTLA-4 inhibitor tremelimumab demonstrated an ORR of 43% in the 7 patients included with previously treated metastatic TNBC [49]. All patients with TNBC had ongoing response of at least 10 mos at the time of data cutoff. Toxicity was consistent with previous reports of dual checkpoint blockade—nearly every patient had some degree of hepatitis during treatment, but no grade 4/5 adverse events were documented. The multicohort phase II DART trial evaluated the combination of ipilimumab and nivolumab in 17 patients with metaplastic breast cancer, which were predominantly triple-negative [50]. Responses were seen in 3 patients, all ongoing for at least 11 mos; hepatotoxicity and fatigue were the most common toxicities. Lymphocyte activating gene-3 (LAG-3) is an emerging immunotherapy target, which acts synergistically with PD-1 as a negative regulator of T-cell activity [51]. The combination of IMP701 (LAG525), an anti-LAG-3 antibody, with or without the anti-PD-1 antibody spartalizumab, was evaluated in patients with several solid tumors. No responses were seen in any disease with LAG525 alone, although 2 of 5 patients with TNBC responded to combination therapy [52]. REGN3767 is another LAG-3 directed antibody, and is currently under investigation in combination with cemiplimab as neoadjuvant treatment in one of the arms of the I-SPY2 trial. In an alternative approach, IMP321 (eftilagimod alpha) consists of the extracellular domain of LAG-3 fused to the Fc portion of the human immunoglobulin which, instead of functioning as a LAG-3 antagonist, activates antigen presenting cells as an agonist of the class II major histocompatibility complex. IMP321 was evaluated in combination with weekly paclitaxel in a phase I/II trial of 30 metastatic breast cancer patients who had not yet received chemotherapy, 4 of whom were ER negative [53]. An ORR of 50% was reported (not specified for the ER negative subgroup), and toxicities appeared to be consistent with paclitaxel monotherapy, but further study in a randomized trial for hormone receptor positive disease yielded negative results [54]. Therapies targeting other immune checkpoints, including VISTA [55], TIGIT [56], 4-1BB [57], and OX40 [58] are also under investigation.

PARP inhibitors have well documented activity in *BRCA*-associated metastatic breast cancer, but can also upregulate PD-L1 on tumor cells, inhibiting T-cell mediated tumor death [59]. Combining checkpoint inhibitors with PARP inhibitors may therefore restore immune recognition of breast cancer in PARP inhibitor treated individuals. Preclinical models have demonstrated synergy between PARP inhibitors and anti–PD-1/PD-L1 antibodies, regardless of *BRCA* mutation status and PD-L1 expression [59]. The single arm phase II TOPACIO/KEYNOTE-162 trial evaluated the PARP inhibitor niraparib in combination with pembrolizumab in metastatic TNBC [60]. Patients with up to three prior lines of chemotherapy were eligible, but could not have progressed on prior platinum chemotherapy. In 47 efficacy-evaluable patients, the ORR was 21% and the disease control rate (DCR) at 9 weeks was 49%; responses were particularly enriched in those with mutations in *BRCA1/2* (ORR 47%) and in those with PD-L1 CPS ≥ 1 (ORR 32%). No unexpected safety signal was seen with the combination—4% of patients had grade 3 immune-related adverse events (irAEs), no higher-grade immune toxicities were seen. The MEDIOLA phase I/II trial evaluated the PARP inhibitor olaparib in combination with durvalumab in HER2-negative, *BRCA*-associated metastatic breast cancer [61]. Patients were required to have received anthracycline or taxane chemotherapy (in the early or advanced setting), but no more than two prior lines of treatment for advanced breast cancer. Patients who progressed on prior platinum agents or had been treated with platinum in the past 12 months were excluded. The 17 patients with TNBC had an ORR of 58.8% with a median duration of response of over 12 mos. The response rates of patients with *BRCA* mutations in these trials mirror the ORR in OlympiAD (54.7%) and EMBRACA (61.8%), so it is uncertain if significant synergy exists with this combination [3, 4]. However, multiple randomized trials are ongoing to further evaluate PARP inhibitor and checkpoint blockade in TNBC, including evaluation as maintenance therapy in the phase II/III KEYLYNK-009 trial (NCT04191135) [62] and a randomized phase II trial of olaparib with or without atezolizumab for patients with a germline *BRCA* mutation (NCT02849496) [63].

Immunotherapy has been evaluated in combination with multiple other cancer therapies in early phase clinical trials. Lenvatinib has demonstrated immune modulatory effects through reduction in tumor associated macrophages and increased type I interferon signaling [64]. The ongoing phase II LEAP-005 trial has reported preliminary efficacy of lenvatinib and pembrolizumab with an ORR of 29% in 31 patients with TNBC [65]. Specific AKT inhibitors may also contribute to favorable changes in the tumor microenvironment through reduction in regulatory T cells [66]. In an expanded phase 1b trial of atezolizumab, a taxane, and the AKT inhibitor ipatasertib in 114 patients with untreated TNBC, an ORR of 54% was seen with a median PFS of 7.2 mos in the overall as well as the PD-L1 negative population [67]. Bevacizumab is theorized to prime the tumor microenvironment through normalization of tumor vasculature, and a multicenter phase II study of bevacizumab, nivolumab, and paclitaxel in untreated TNBC demonstrated an ORR of 59% and median PFS of 8.1 mos [68]. Although the preclinical rationale for these combinations is sound, the preliminary results are comparable to other trials of checkpoint blockade and taxane therapy in untreated TNBC. A number of intratumoral injections that directly alter the microenvironment have reported preliminary data in advanced TNBC in combination with checkpoint blockade, including oncolytic virus talimogene laherparepvec [69] and the DNA plasmid tavokinogene telseplasmid [70], but further data are needed to draw firm conclusions on efficacy.

Not all attempts at improving immune response have had positive results. Histone deacetylase (HDAC) inhibitors have been evaluated in numerous settings within oncology, and theoretically enhance cancer antigen expression and modulate immunosuppressive cells [71]. However, a randomized phase II trial failed to demonstrate benefit of the addition of the HDAC inhibitor entinostat to atezolizumab in a population of previously treated TNBC patients [72]. The phase II COLET trial of the MEK inhibitor cobimetinib, hypothesized to increase sensitivity to taxanes and PD-L1 inhibitors, demonstrated no benefit from the addition of atezolizumab to cobimetinib and a taxane [73]. Nonetheless, a number of novel treatment strategies have now shown promising preliminary efficacy that will extend the benefit of immunotherapy to a greater proportion of patients with TNBC.

### Early-stage triple-negative breast cancer

The promising efficacy of checkpoint inhibitors in the metastatic setting has prompted further exploration of immunotherapy as part of curative intent therapy for early-stage TNBC (Table 3). In the metastatic setting, response to immunotherapy is higher in patients with fewer lines of prior treatment, and use in the early-stage disease provides an opportunity to capitalize on the more favorable microenvironment of untreated patients. Furthermore, even with the addition of carboplatin to neoadjuvant therapy, half of patients will have residual disease [74] which is associated with a particularly poor prognosis—as nearly one third of such patients will recur despite additional adjuvant treatment [75].

**Table 3:** Randomized clinical trials of neoadjuvant/adjuvant immunotherapy for triple-negative breast cancer

Trial	Patient population	Treatment arms	Subgroups	Sample size		pCR rate (%)	
				IN	CT	IN	CT
NCT01042379^a^ I-SPY2	Tumor ≥ 2.5 cm	T + pembro × 4 → AC × 4	ITT	29	85	60	22
		T × 4 → AC × 4					
		T + pembro × 4 → pembro × 4	ITT	73^b^	295^b^	27	27
		T × 4 → AC × 4					
		olaparib + durvalumab + T × 4 → AC × 4	ITT	22	142	47	27
		T × 4 → AC × 4					
		T + pembro × 4 + SD-101 → AC × 4	ITT	29	147	44	28
		T × 4 → AC × 4					
NCT03036488	T1cN1-2 or	T + carbo + pembro → AC/EC +	ITT	784	390	63.0	55.6
		pembro × 4 → surgery → pembro	PD-L1 CPS ≥ 1	560^c^	158^c^	67.1	58.3
KEYNOTE-522	T2-4N0-2		PD-L1 CPS < 1	109^c^	56^c^	47.7	37.3
		T + carbo + placebo → AC/EC +	Lymph Node +	349^c^	167^c^	62.8	49.7
		placebo × 4 → surgery → placebo	Lymph Node –	320^c^	166^c^	65.3	59.6
NCT02685059	Tumor ≥ 2 cm	*nab*-T + durvalumab × 4	ITT	88	86	53.4	44.2
GeparNuevo		→ EC + durvalumab × 4	PD-L1 IC/TC ≥ 1%	69	69	58.0	50.7
			PD-L1 IC/TC < 1%	9	11	44.4	18.2
		*nab*-T × 4 + placebo × 4	Window	59	58	61.0	41.4
		→ EC × 4 + placebo × 4	Concurrent	29	28	37.9	50.0
NCT02620280	T1cN1 + or T3N0 +	*nab*-T + carboplatin + atezolizumab × 8	ITT	142	138	43.5	40.8
	Unilateral IDC	→ surgery → AC/EC/FEC × 4	PD-L1 IC ≥ 1%	77	79	51.9	48.0
		*nab*-T + carboplatin × 8	PD-L1 IC < 1%	65	59	32.2	32.3
NeoTRIPaPD-L1							
	High Ki-67 or Grade	→ surgery → AC/EC/FEC × 4					
NCT03197935	Unilateral	*nab*-T × 12 + atezolizumab × 6	ITT	165	168	58	41
		→ EC × 4 + atezolizumab × 4	PD-L1 IC ≥ 1%	77	75	69	49
			PD-L1 IC < 1%	88	93	48	34
		*nab*-T × 12 + placebo × 6	Lymph Node +	56	72	57	31
IMpassion031	tumor > 2 cm	→ EC × 4 + placebo × 4	Lymph Node –	109	96	58	49

Intervention arm regimen listed followed by control arm for each trial

*pCR* pathologic complete response, *IN* intervention, *CT* Control, *T* paclitaxel, *A* doxorubicin, *C* cyclophosphamide, *E* epirubicin, *F* 5-fluorouracil, *ITT* intention to treat, *PD-L1* programmed death-ligand 1, *IDC* invasive ductal carcinoma

^a^Data from the triple-negative subgroup of included patients is listed

^b^Sample size listed for overall population as breakdown of population by HR + /HER2- status not published

^c^Whereas the ITT pCR rates are reported for all patients as from the third interim analysis, responses in subgroups were only available from the second interim analysis of 1002 patients

Four approaches combining immunotherapy with standard neoadjuvant chemotherapy have been explored in the phase 2 adaptively randomized I-SPY2 trial. The unique design of I-SPY2 allows for the comparison of multiple investigational treatments to a continuously enrolling contemporary control arm, which for TNBC patients consisted of 12 doses of weekly paclitaxel followed by 4 cycles of doxorubicin and cyclophosphamide (AC) [76, 77]. Patients are randomized to a treatment arm based on the Bayesian probability of the investigational treatment being superior to control in the primary outcome of pCR, and enrollment for an investigational arm ends when at least 60 patients are enrolled and the predicted probability of success in a confirmatory phase 3 trial reaches at least 85%. The first immunotherapy arm in I-SPY2 evaluated 4 cycles of pembrolizumab concurrently with weekly paclitaxel, followed by AC (pembro4) [78]. The 29 patients with TNBC had a pCR rate of 60% with pembrolizumab compared to 22% in 85 who receiving standard chemotherapy. Common irAEs included pruritus (31.9%), hypothyroidism (10.1%), and adrenal insufficiency (8.7%). An exploratory biomarker study did not find an association between pCR and tumor cell PD-L1 positivity or PD-L1 gene expression [79]; conversely Th1 cell, B cell, and dendritic cell gene signatures were predictive of pCR, even when controlling for response to standard chemotherapy and HR status. A subsequent arm evaluated de-escalation of therapy, treating patients with 4 cycles of pembrolizumab + weekly paclitaxel followed by an additional 4 cycles of pembrolizumab alone (pembro8-noAC) [80]. After three patients progressed while receiving pembrolizumab monotherapy, enrollment to the arm was halted and investigators had the option to move to surgery after pembrolizumab with weekly paclitaxel or administer pembrolizumab concurrently with AC prior to surgery. Patients randomized to this arm who received anthracyclines in the neoadjuvant setting were considered non-pCR for the efficacy analysis. The TNBC patients enrolled on this arm had a pCR rate of 27%, which was identical to the rate of pCR in the contemporary control group. Although pembro8-noAC did not meet the efficacy endpoint, the comparable pCR rates suggest that immunotherapy may have a role in patients who are unable to tolerate or receive anthracyclines.

The I-SPY2 trial also evaluated 4 cycles of pembrolizumab along with serial intratumoral injections of the toll like receptor 9 (TLR9) agonist SD-101 concurrent with weekly paclitaxel, and followed by standard AC. Pre-clinical models have shown that SD-101 can overcome resistance in anti-PD-1 non-responders, promoting infiltration of activated T-cells and type I interferon signaling [81]. The pCR rate was 44% in 29 patients with TNBC treated with SD-101 and pembrolizumab, versus 28% in contemporary controls [82]. Although the probability that this regimen produced superior responses compared to control treatment exceeded 97%, the predicted probability of success in a phase 3 trial did not reach the pre-specified threshold of 85%. Given the previous success of the pembro4 arm, these results raise questions as to the additive benefit of SD-101, although further analysis of this arm is ongoing. Finally, the I-SPY2 trial evaluated the administration of durvalumab, olaparib, and weekly paclitaxel, followed by standard AC [83]. A total of 73 patients received durvalumab and olaparib, including 21 with TNBC. The pCR rate was 47% in TNBC patients compared to 27% in controls, and response was associated with a number of mRNA immune signatures in both the investigational and control arms, although no signature specifically predicted benefit from the addition of immunotherapy in TNBC patients. Notable irAEs included adrenal insufficiency (9.3%, including both primary and secondary cases of AI, all grades) and colitis (7%, grade 3 +). Although cross trial comparisons are limited, the pCR benefit was similar to other trials of neoadjuvant immunotherapy, and the benefit of adding olaparib to neoadjuvant regimens remains uncertain.

The randomized, double-blind phase II GeparNuevo trial evaluated the addition of durvalumab to neoadjuvant chemotherapy in those with early-stage TNBC and provided unique insights into the impact of treatment on the immune microenvironment [84]. Durvalumab/placebo was administered every 4 weeks in conjunction with 12 doses of weekly *nab*-paclitaxel followed by 4 doses of epirubicin and cyclophosphamide. The first 117 patients also received a dose of durvalumab/placebo 2 weeks prior to initiation of chemotherapy (termed the window-phase treatment), but this was halted due to ethical concerns regarding the delay in chemotherapy initiation for those receiving placebo. While there was a numerical improvement in the pCR rate in the durvalumab group vs the control group, this difference was not statistically significant (53.4% vs 44.2%, *p* = 0.287). However, receipt of durvalumab was associated with improved distant disease-free survival (HR 0.37; 95% CI: 0.15–0.87, *p* = 0.0148) and OS (HR 0.26; 95% CI: 0.09–0.79, *p* = 0.0076) [85]. Pre-treatment stromal TIL levels were associated with response in both arms. On serial biopsies during the window phase, intratumoral TILs from pre-treatment to 2 weeks post-treatment was seen in both the durvalumab and placebo arms, but increasing TILs was associated with response only in the durvalumab arm. This is consistent with studies of checkpoint blockade in melanoma [86], suggesting that immunotherapy efficacy is dependent on immune cell recruitment. PD-L1 TC positivity was associated with durvalumab but not chemotherapy response; conversely, PD-L1 IC positivity was associated with chemotherapy but not durvalumab response.

The multicenter open-label phase III NeoTRIPaPDL1 study randomized 280 patients with locally advanced or early high-risk TNBC to neoadjuvant carboplatin and *nab*-paclitaxel, with or without atezolizumab. Patients then underwent surgery, followed by adjuvant AC per investigator’s discretion. The primary outcome of 5-year event-free survival (EFS) is immature, but the trial did not meet the secondary endpoint of pCR in a recent report. The pCR rate was similar with atezolizumab compared with controls (43.5% vs 40.8%, respectively, *p* = 0.66), even when selecting for PD-L1 IC + tumors (51.9% vs 48.0%, statistical significance not reported). Safety signals were consistent with past trials. Interestingly, TIL levels were significantly higher in the chemotherapy arm, perhaps leading to amplified responses in the chemotherapy arm and contributing to the diminutive benefit of immunotherapy [87].

More encouraging results were seen with the double-blind, randomized phase III IMpassion031, which compared the chemotherapy regimen of *nab*-paclitaxel for 12 weeks followed by 8 weeks of AC, with atezolizumab or placebo administered every 2 weeks [88]. Patients with stage II-III TNBC were randomized 1:1 to the two treatment arms, stratified by stage and PD-L1 status. Co-primary endpoints were pCR in the ITT and PD-L1 IC positive subgroups. With a total of 333 enrolled patients, pCR was significantly improved with atezolizumab in the overall population (58% vs 41%, *p* = 0.0044), but did not cross the significance boundary in the PD-L1 IC positive population (69% versus 49%, *p* = 0.021). The benefit of atezolizumab was higher in lymph node positive patients, with a 27% improvement in pCR rates; compared to lymph node negative patients, where only a 9% improvement in pCR was seen (significance not formally tested). Rates of hypothyroidism were 7% with atezolizumab, and no adrenal insufficiency or hypophysitis was observed. The contrasting results with NeoTRIPaPDL1 may suggest synergy between anthracyclines and immunotherapy (as anthracyclines were only given post-operatively in NeoTRIPaPDL1). This is also supported by the higher response rates seen after induction doxorubicin in patients with metastatic disease in the TONIC trial. Long-term follow-up data from NeoTRIPaPDL1 may clarify if the benefit of adjuvant anthracyclines is more prominent in patients who received neoadjuvant immunotherapy.

Prior to evaluation of pembrolizumab as part of neoadjuvant treatment in a phase III trial, the multi-cohort KEYNOTE-173 trial evaluated 6 different chemoimmunotherapy regimens in 60 patients with TNBC and T2-4N0 disease, or T1c disease with lymph node involvement [89]. Chemotherapy included 6 dosing schedules of a taxane with or without carboplatin followed by AC, and all patients received pembrolizumab for nine cycles, beginning three weeks prior to planned chemotherapy. The taxane regimens included weekly *nab*-paclitaxel at 125 mg/m^2^ (cohorts A, C, and D) or 100 mg/m^2^ (cohort B), or weekly paclitaxel 80 mg/m^2^ (cohorts E and F). The carboplatin regimens included every 3 week treatment at an AUC of 6 (cohort B) or 5 (cohorts C and E), or weekly treatment at an AUC of 2 (cohorts D and F). Overall pCR rate was 60%, and the pCR rate was 60% in patients who had PD-L1 CPS ≥ 1, compared to 40% in those with PD-L1- disease. Although pCR rates did not vary between those who did (cohorts B–F) and did not (cohort A) receive carboplatin, EFS at 12 mos was 98% in patients who received carboplatin versus 80% in those who did not. Pre-treatment stromal TILs were also associated with response. The most common irAEs included thyroid disorders (13%), colitis (3%), hypersensitivity (3%), and rash (3%).

The impressive EFS outcomes with platinum and immunotherapy based treatment in KEYNOTE-173 informed the design of the phase III randomized double-blind KEYNOTE-522 trial, which evaluated pembrolizumab versus placebo plus standard neoadjuvant chemotherapy in individuals with early-stage TNBC [90]. Patients were randomized in a 2:1 fashion to receive pembrolizumab vs placebo plus 12 weeks of carboplatin and paclitaxel followed by 12 weeks of an anthracycline plus cyclophosphamide; pembrolizumab/placebo was administered every 3 weeks concurrently with neoadjuvant chemotherapy. Postoperatively, patients received up to 9 cycles of pembrolizumab/placebo. Although the first interim analysis demonstrated a significant improvement in pCR and EFS, the improvement in pCR with immunotherapy decreased from 13.6% to 7.5% by the third interim analysis (63.0% vs 55.6%, statistical testing not formally performed). Nonetheless, at the fourth interim analysis, a meaningful and statistically significant improvement in EFS was documented (3-year EFS 84.3% versus 76.2%, *p* = 0.00031) [91]. This led to the FDA approval of pembrolizumab in combination with neoadjuvant chemotherapy (with continuation as a single agent after surgery) for high-risk early-stage TNBC. Responses were similar in the PD-L1 CPS ≥ 1 and < 1 subgroups at first interim analysis, although unpublished data from the third interim analysis suggest benefit is enriched in the PD-L1 CPS ≥ 20 population (HR for EFS 0.36; 95% CI: 0.16–0.79, statistical testing not performed) [92]. Axillary lymph node involvement was associated with a more substantial pCR benefit with the addition of pembrolizumab, although the EFS benefit with immunotherapy was similar regardless of lymph node involvement at the third interim analysis. Observed irAEs included hypothyroidism (15.0%), hyperthyroidism (5.2%), severe skin toxicity (5.8%), and primary and secondary adrenal insufficiency (4.5%) [92].

### The future of immunotherapy in triple-negative breast cancer

With the FDA approval of pembrolizumab, immunotherapy has finally emerged at the forefront of treatment of TNBC. Atezolizumab had initially shown promise for the treatment of advanced TNBC, but this approval has been withdrawn given the absence of benefit in the IMpassion131 trial. Further analysis of IMpassion131 will be essential to determine if confounders, such as TILs, were imbalanced across the two arms, similar to what was seen in the NeoTRIPaPDL1 trial. Conversely, pembrolizumab has approval in advanced TNBC with PD-L1 CPS ≥ 10, in combination with paclitaxel, *nab*-paclitaxel, or gemcitabine plus carboplatin, with similar outcomes seen regardless of choice of chemotherapy. Studies have also identified that a small subset of patients may benefit from checkpoint blockade monotherapy, with superior quality of life outcomes in the CPS ≥ 10 population in KEYNOTE-119, and with a provocative exploratory analysis suggesting a survival advantage for immunotherapy over chemotherapy in patients with CPS ≥ 20. Checkpoint blockade monotherapy may play an evolving role in select patients with advanced TNBC, but currently approval is limited to those with TMB ≥ 10 muts/Mb or MSI-H status.

In the curative setting, multiple studies have confirmed a pCR benefit with the addition of immunotherapy to neoadjuvant chemotherapy regimens for early-stage TNBC. Emerging data from GeparNuevo and KEYNOTE-522 suggest that the addition of immunotherapy also reduces disease recurrence. Based on the results of KEYNOTE-522, pembrolizumab is now approved as part of neoadjuvant treatment and as single agent adjuvant treatment for high-risk early-stage TNBC. Ongoing trials such as IMpassion030 (NCT03498716) will confirm if this benefit extends to patients receiving purely adjuvant chemotherapy, who might conceivably have a lower volume of immunogenic tumor antigens. Furthermore, the value of adjuvant immunotherapy alone in patients with residual disease after neoadjuvant chemotherapy will be addressed by the SWOG 1418 trial (NCT02954874).

Despite successes in both advanced and early-stage disease, PD-1/PD-L1 inhibitors have not proven to be the panacea for TNBC, improving outcomes in a fraction of patients. Novel checkpoint blockade targeting LAG-3 [51], VISTA [55], and TIGIT [56], costimulatory agonists of 4-1BB [57] and OX40 [58], as well as combinations with checkpoint inhibitors may lead to an increasing proportion of responders. Immune potentiating and microenvironment altering strategies—including small-molecule inhibitors, PARP inhibitors, and injectable agents such as oncolytic viruses, DNA plasmids, and toll like receptor agonists [93]—may induce immune responses in ‘cold’ tumors. A number of engineered cellular therapy products are under development with an increasing understanding of TNBC antigenic targets [94]. Identifying the most effective chemotherapy agent and sequence for combination with immunotherapy as well as incorporating radiotherapy strategies may further overcome resistance.

With the number of therapeutic approaches exponentially expanding, it will become increasingly important to apply precision medicine approaches to match patients with effective treatments. Evolving biomarkers—including gene expression signatures and multiplex immunofluorescence (which allows for spatial characterization of the tumor microenvironment)—may better match patients with effective treatment strategies [95]. There is a particularly acute need to identify patients who benefit most from neoadjuvant immunotherapy, as predictive biomarkers in the metastatic setting such as PD-L1 have not translated to early-stage disease, and immunotherapy may be associated with irreversible, lifelong irAEs in patients with curable breast cancer. Precise models of response to neoadjuvant chemotherapy may play a role, identifying patients likely to have a good outcome without the addition of immunotherapy [96, 97]. Novel, data-intensive and computational techniques are needed to approach the increasing amount of data available from each patient to better understand response to immunotherapy, as it relates to the complex interplay between tumor gene expression, histologic and imaging findings, and even host related factors such as microbiome diversity. Large public repositories, such as The Cancer Genome Atlas [98] and the ISPY1 trial [99] have fueled big-data approaches to breast cancer biology, but as clinical trials are increasingly digitized [100], emphasis must be placed on prompt sharing of patient level correlative data to truly democratize immunotherapy biomarker discovery. Nonetheless, the excitement surrounding immunotherapy is palpable, contributing to the steady progress in ameliorating the historically poor prognosis of triple-negative breast cancer.
